# Supplementary material for: Machine Learning Based Lens-Free Shadow Imaging Technique for Field-Portable Cytometry
Source: Biosensors (Basel). 2022 Feb 27;12(3):144. doi: 10.3390/bios12030144 (PMC8946550; doi:10.3390/bios12030144)
Supplement: Supplementary file 1 [file biosensors-12-00144-s001.zip › biosensors-1598249-supplementary.pdf]

biosensors

MDPI

Supplementary Information

Machine Learning Based Lens-Free Shadow Imaging Technique for Field-Portable Cytometry

Rajkumar Vaghashiya [1,†], Sanghoon Shin [2,†], Varun Chauhan [1], Kaushal Kapadiya [1], Smit Sanghavi [1], Sungkyu Seo [2,*] and Mohendra Roy [3,*]

1 Department of Computer Engineering, Pandit Deendayal Energy University, Gandhinagar 382007, India; rajkumar.vce16@sot.pdpu.ac.in (R.V.); varun.cce16@sot.pdpu.ac.in (V.C.); kaushal.kce16@sot.pdpu.ac.in (K.K.); smit.sce16@sot.pdpu.ac.in (S.S.)
2 Department of Electronics and Information Engineering, Korea University, Sejong 30019, Korea; ghost10s@korea.ac.kr
3 Department of Information and Communication Technology, Pandit Deendayal Energy University, Gandhinagar 38207, India
* Correspondence: sseo@korea.ac.kr (S.S.); mohendra.roy@ieee.org (M.R.); Tel: +82-44-860-1427 (S.S.); +91-79-2327-5483 (M.R.); Fax: +82-44-860-1585 (S.S.); +91-79-2327-5030 (M.R.)
† These authors contributed equally to this study.

Citation: Vaghashiya, R.; Shin, S.; Chauhan, V.; Kapadiya, K.; Sanghavi, S.; Seo, S.; Roy, M. Machine Learning Based Lens-Free Shadow Imaging Technique for Field-Portable Cytometry. *Biosensors* **2022**, *12*, 144. https://doi.org/10.3390/bios12030144

Received: 30 January 2022
Accepted: 25 February 2022
Published: 27 February 2022

Publisher's Note: MDPI stays neutral with regard to jurisdictional claims in published maps and institutional affiliations.

Copyright: © 2022 by the authors. Licensee MDPI, Basel, Switzerland. This article is an open access article distributed under the terms and conditions of the Creative Commons Attribution (CC BY) license (https://creativecommons.org/licenses/by/4.0/).

1. Convolutional Neural Network (CNN) workflow

Network Architecture			Output dimensions
0-Input	:	N, 50x50 (batch size, input image)	
1-Conv2D	:	64, 3x3,	48x48, Relu
2-Conv2D	:	32, 3x3,	46x46, Relu
3-MaxPool2D	:	32, 3x3,	15x15
4-Conv2D	:	16, 3x3,	13x13, Relu
5-MaxPool2D	:	16, 3x3,	4x4
6-Flatten	:	16, 4x4,	256
7-FC	:		128, Relu
8-FC	:		64, Relu
9-FC	:		6, Softmax

Here, FC represents Fully Connected layers in the CNN architecture.

Forward Propagation

Step1:

Given an input image I (50x50 single greyscale image) to be convolved with 'p' kernels, the 2D convolution operation C is defined as

$$C_p^1 = f(\, I * k_{1,p}^1 \; + b_p^1\,);\ \text{p=1 to 64}$$

where $k_{1,p}^1$ – "1" in the superscript denotes the hierarchal order of the convolution operation

"1, p" in the subscript denotes 1 input channel, p^{th} output channel

b_p^1 – bias corresponding to each of the p kernels

* represents the convolution (element-wise product followed by addition) between the input and the kernel

f represents Relu activation, applied to the output of the convolution operation

Step2:

$$C_q^2 = f(\, \sum_p C_p^1 * k_{p,q}^2 \; + b_q^2\,);\ \text{p=1 to 64; q= 1 to 32}$$

Step3:

$$M_q^1(i,j) = \max\big(\, C_q^2\,[\,3(\mathrm{i}-1)+1 \;:\; 3(\mathrm{i}-1)+3\,, 3(\mathrm{j}-1)+1 \;:\; 3(\mathrm{j}-1)+3\,]\big)$$

i, j=1 to 15; q= 1 to 32;

Step4:

$$C_r^3 = f(\, \sum_q M_q^1 * k_{q,r}^3 \; + b_r^3\,);\ \text{q= 1 to 32;}\quad \text{r = 1 to 16}$$

Step5:

$$M_r^2(i,j) = \max(\, C_r^3\,[\,3(\mathrm{i}-1)+1 \;:\; 3(\mathrm{i}-1)+3\,, 3(\mathrm{j}-1)+1 \;:\; 3(\mathrm{j}-1)+3\,])$$

i, j=1 to 4; r= 1 to 16;

Step6:

$$g \;= G(\,\{M_r^2\}\,; \text{r = 1 to 4}\;);\quad \text{(flatten/vectorization)}$$

The rest of the steps for the fully connected layers are the same as in Feedforward Multi-Layer Perceptron (MLP).

Backward Propagation

Let L be the loss at the end of the epoch during the training phase.

All the equations for backpropagation for the fully connected layers are the same as in MLP till the FC-7 layer.

Step1:

Those computed gradients from layer FC-7 simply need to be vectorized into matrix 4x4x16

Reshape output error vector Δg (256x1) into (4x4x16)

$$\{\Delta M_r^2\}_{\mathrm{r=1\ to\ 4}} = G^{-1}(\Delta g); \quad \text{(flatten/vectorization)}$$

Since it is a max-pool operation, it doesn't involve any parameters, hence the stored mask in the forward pass is used as a reference for the backpropagation update path, giving ΔC_r^3.

Step2: (Inverse of step 4 in the forward path)

Calculating $\Delta k_{q,r}^3$:

$$\Delta k_{q,r}^3(u,v) = \frac{\partial L}{\partial k_{q,r}^3(u,v)} = \sum_i^{13}\sum_j^{13} \frac{\partial L}{\partial C_r^3(i,j)} \frac{\partial C_r^3(i,j)}{\partial k_{q,r}^3(u,v)}$$

$$= \sum_i^{13}\sum_j^{13} \Delta C_r^3(i,j) \,.\frac{\partial C_r^3(i,j)}{\partial t} \frac{\partial \mathrm{t}}{\partial k_{q,r}^3(u,v)}; t = (\sum_q M_q^1 * k_{q,r}^3 + b_r^3)$$

$$= \sum_i^{13}\sum_j^{13} \Delta C_r^3(i,j) \,.f' . \frac{\partial\left(\sum_q M_q^1 * k_{q,r}^3 + b_r^3\right)}{\partial k_{q,r}^3(u,v)}$$

$$= \sum_i^{13}\sum_j^{13} \Delta C_r^3(i,j) \,.f' . \; M_q^1(i-u, j-v)$$

Let $\Delta C_{r,f}^3(i,j) = \Delta C_r^3(i,j) \,.f'$

Hence, we get a deconvolution as follows:

$$\Delta k_{q,r}^3(u,v) = \sum_i^{13}\sum_j^{13} M_q^1(i-u, j-v) \,.\Delta C_{r,f}^3(i,j)$$

Here, ${M_q^1}^{inv}$ which is equivalent to convolving with the inverse of M_q^1 i.e. rotating M_q^1 by 180° (flipped)

$$\Delta k_{q,r}^3 = {M_q^1}^{inv} * \Delta C_{r,f}^3$$

Calculating ΔM_q^1:

Similarly, Following the above process for ΔM_q^1, we get similar deconvolution:

$$C_r^3 = f(\textstyle\sum_q M_q^1 * k_{q,r}^3 + b_r^3);$$

$$\Delta M_q^1(i,j) = \frac{\partial L}{\partial M_q^1(i,j)}$$

$$\Delta M_q^1 = \sum_r^{15} \Delta C_{r,f}^3 * k_{q,r}^{3\ inv}$$

Calculating Δb_r^3:

$$\Delta b_r^3 = \frac{\partial L}{\partial b_r^3} = \sum_i^{13}\sum_j^{13} \frac{\partial L}{\partial C_r^3(i,j)} \frac{\partial C_r^3(i,j)}{\partial b_r^3}$$

$$= \sum_i^{13}\sum_j^{13} \Delta C_r^3(i,j) \,.\, \frac{\partial C_r^3(i,j)}{\partial t} \frac{\partial \mathrm{t}}{\partial b_r^3}; t = (\sum_q M_q^1 * k_{q,r}^3 + b_r^3)$$

$$= \sum_i^{13}\sum_j^{13} \Delta C_r^3(i,j) \,.\, f' . \ \frac{\partial \left(\sum_q M_q^1 * k_{q,r}^3 + b_r^3\right)}{\partial b_r^3}$$

$$= \sum_i^{13}\sum_j^{13} \Delta C_r^3(i,j) \,.\, f'$$

$$\Delta b_r^3 = \sum_i^{13}\sum_j^{13} \Delta C_{r,f}^3 (i,j)$$

<u>Step3: (Inverse of step 3 in the forward path)</u>

Since it is a max-pool operation, it doesn't involve any parameters, the stored mask in the forward pass is used as a reference for backpropagation. Updates from ΔM_q^1 will be propagated into respective ΔC_q^2.

<u>Step4: (Inverse of step 2 in the forward path)</u>

Calculating $\Delta k_{p,q}^2$:

$$\Delta k_{p,q}^2 (u,v) = \frac{\partial L}{\partial k_{p,q}^2 (u,v)} = \sum_i^{46}\sum_j^{46} \frac{\partial L}{\partial C_q^2 (i,j)} \frac{\partial C_q^2 (i,j)}{\partial k_{p,q}^2 (u,v)}$$

$$= \sum_i^{46}\sum_j^{46} \Delta C_q^2 (i,j) \,.\, \frac{\partial C_q^2 (i,j)}{\partial t} \frac{\partial \mathrm{t}}{\partial k_{p,q}^2 (u,v)}; t = (\sum_p C_p^1 * k_{p,q}^2 + b_q^2)$$

$$= \sum_i^{46}\sum_j^{46} \Delta C_q^2 (i,j) \,.\, f' . \ \frac{\partial \left(\sum_p C_p^1 * k_{p,q}^2 + b_q^2\right)}{\partial k_{p,q}^2 (u,v)}$$

Equivalently,

$$\Delta k_{p,q}^{2} = {C_p^1}^{inv} * \Delta C_{q,f}^{2}$$

Calculating ΔC_p^1:

Similarly, Following the above process for ΔM_q^1, we get similar deconvolution:

$$\Delta C_p^1(i,j) = \frac{\partial L}{\partial C_p^1(i,j)}$$

$$\Delta C_q^1 = \sum_{q}^{48} \Delta C_{q,f}^2 * {k_{p,q}^2}^{inv}$$

Calculating Δb_q^2:

$$\Delta b_q^2 = \frac{\partial L}{\partial b_q^2} = \sum_{i}^{46}\sum_{j}^{46} \frac{\partial L}{\partial C_q^2(i,j)} \frac{\partial C_q^2(i,j)}{\partial b_q^2}$$

$$\Delta b_q^2 = \sum_{i}^{46}\sum_{j}^{46} \Delta C_{q,f}^2(i,j)$$

Step5: (Inverse Of step 1 in the forward path)

Calculating $\Delta k_{1,p}^1$:

$$\Delta k_{1,p}^1(u,v) = \frac{\partial L}{\partial k_{1,p}^1(u,v)} = \sum_{i}^{50}\sum_{j}^{50} \frac{\partial L}{\partial C_p^1(i,j)} \frac{\partial C_p^1(i,j)}{\partial k_{1,p}^1(u,v)}$$

Equivalently,

$$\Delta k_{1,p}^1 = \mathrm{I}^{inv} * \Delta C_{p,f}^1$$

Calculating Δb_p^1:

$$\Delta b_p^1 = \frac{\partial L}{\partial b_p^1} = \sum_{i}^{50}\sum_{j}^{50} \frac{\partial L}{\partial C_p^1(i,j)} \frac{\partial C_p^1(i,j)}{\partial b_p^1}$$

$$\Delta b_p^1 = \sum_{i}^{50}\sum_{j}^{50} \Delta C_{p,f}^1(i,j)$$

2. Gaussian Noise

The probability density function p(x) for a Gaussian distribution is:

$$p(x) = \frac{1}{\sqrt{2\pi\sigma^2}} e^{\frac{-(x-\mu)^2}{2\sigma^2}}$$

where μ is the mean

σ is the standard deviation

σ^2 is the variance

The generated noise is then added onto the image as

$$\tilde{z} = z + n$$

where z is the noise-free image

n is the noise with Gaussian distribution with desired variance (noise)

3. Traditional denoising methods

Gaussian filtering:

In this, the source image is convolved with the selected Gaussian filter for denoising.

The Gaussian distribution in 1-D has the form:

$$G(x) = \frac{1}{\sqrt{2\pi\sigma^2}} e^{\frac{-x^2}{2\sigma^2}}$$

where σ is the standard deviation of the distribution.

(Here, it is assumed that the distribution has a mean of zero)

Library used – OpenCV (cv2)

Parameters used – kernel size – 3*3 (standard)

Link to the library- https://docs.opencv.org/master/d4/d86/group__imgproc__filter.html#gaabe8c836e97159a9193fb0b11ac52cf1

Average filtering:

Average (or mean) filtering is a method of 'smoothing' images by reducing the amount of intensity variation between neighbouring pixels. The average filter works by moving through the image pixel by pixel, replacing each value with the average value of neighbouring pixels, including itself.

The function smooths an image using the kernel:

$$K = \frac{1}{kernel\ heigth\ \times \text{kernerl width}} \begin{bmatrix} 1 & \cdots & 1 \\ \vdots & \ddots & \vdots \\ 1 & \cdots & 1 \end{bmatrix}$$

Library used – OpenCV (cv2)

Parameters used – kernel size – 3*3

Link to the library- https://docs.opencv.org/master/d4/d86/group__imgproc__filter.html#ga8c45db9afe636703801b0b2e440fce37

Median filtering:

Median filtering is a non-linear method used to remove noise from images. It is widely used as it is very effective at removing noise while preserving edges. It is particularly effective at removing 'salt and pepper' type noise. The image is smoothened using the median filter with the kernel size × kernel size aperture. Each channel of a multi-channel image is processed independently.

Library used – OpenCV (cv2)

Parameters used – kernel size – 3*3

Link to library-https://docs.opencv.org/master/d4/d86/group__imgproc__filter.html#ga564869aa33e58769b4469101aac458f9

Bilateral filtering:

The basic idea underlying bilateral filtering is to do in the range of an image that traditional filters do in its domain. Two pixels can be *close* to one another, that is, occupy the nearby spatial location, or they can be similar to one another, that is, have nearby values, possibly in a perceptually meaningful fashion. Bilateral Filter can reduce unwanted noise very well while keeping edges fairly sharp.

Library used – OpenCV (cv2)

Parameters used – kernel size =3, sigmaColor=3, sigmaSpace=3

Link to the library- https://docs.opencv.org/master/d4/d86/group__imgproc__filter.html#ga9d7064d478c95d60003cf839430737ed

bm3d filtering:

bm3d[1,2] is based on an enhanced sparse representation in the transform-domain. The enhancement of the sparsity is achieved by grouping similar 2D image fragments (e.g. blocks) into 3D data arrays which we call "groups".

Image fragments are grouped based on similarity, but unlike standard k-means clustering and such cluster analysis methods, the image fragments are not necessarily disjoint. This block-matching algorithm is less computationally demanding.

Library used – bm3d

Parameters used – sigma_psd= 15

Link to the library-

https://pypi.org/project/bm3d/ ; http://www.cs.tut.fi/~foi/GCF-BM3D/

4. Comparison of the denoised outputs from various modalities

RBC

$20\mu m$

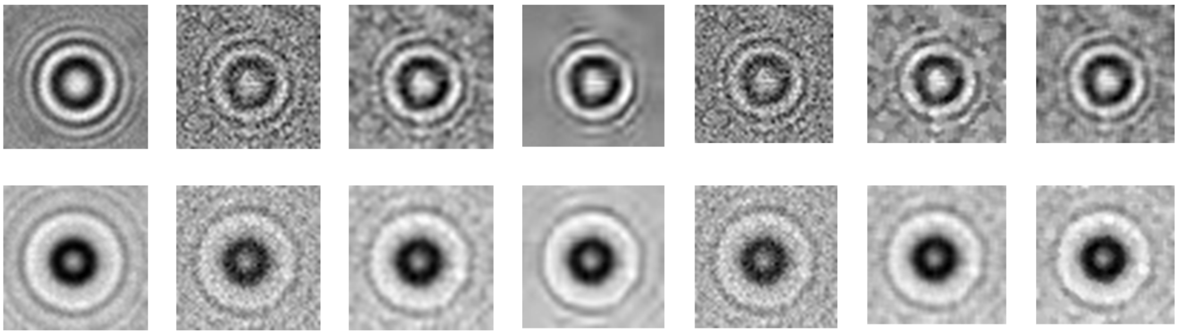

Original | Noisy | Gaussian filtering output | Bm3d filtering output | Bilateral filtering output | Average filtering output | Median filtering output

5. SNR of individual samples of various cell types

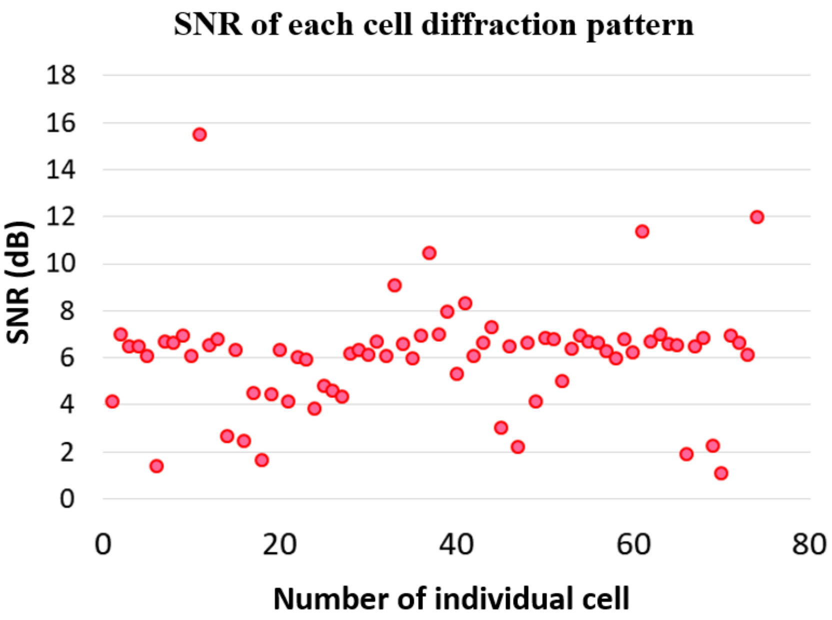

Graph depicting the variation in SNR of individual samples of various cell types denoised by the CNN Autoencoder

6. Grad-CAM and Saliency maps of the individual cell-lines

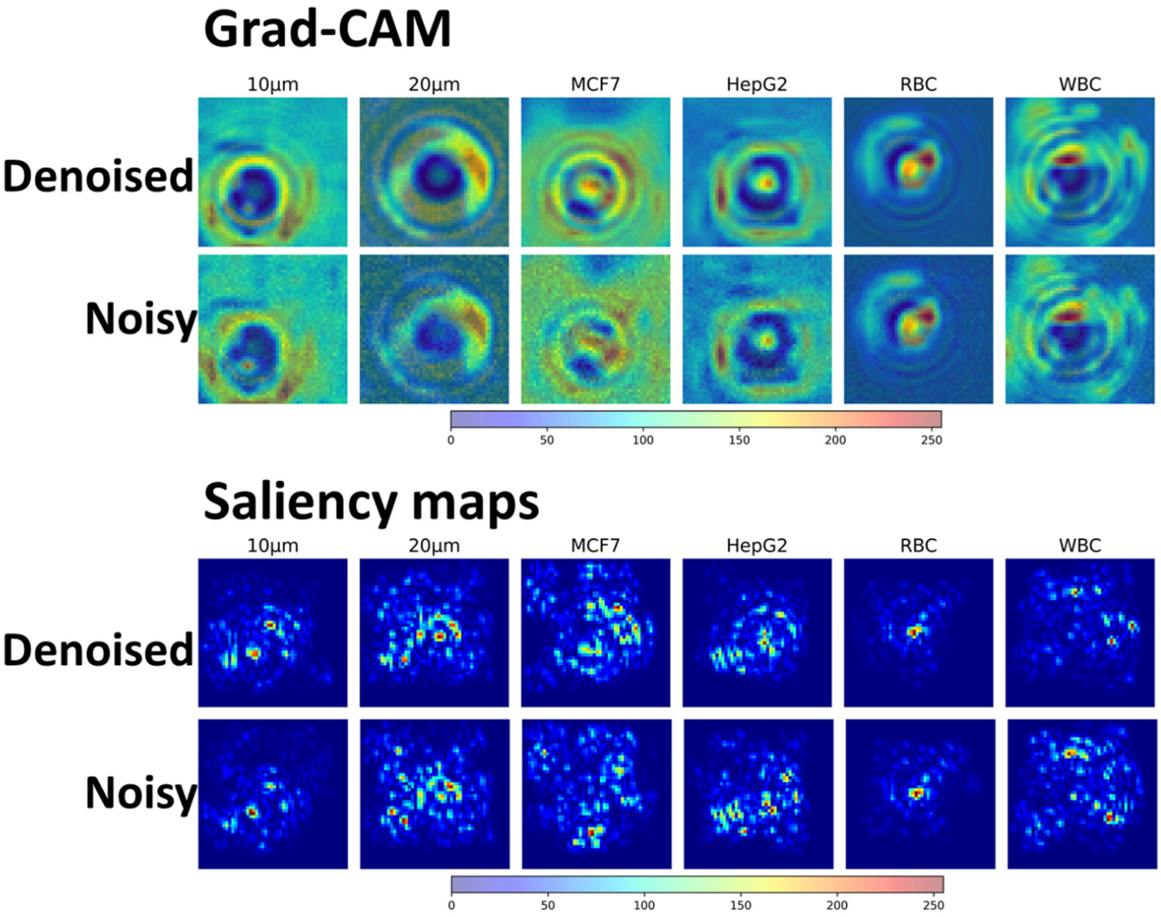

Grad-CAM is a technique to measure the **cumulative activations** from all the layers of the network recorded across the image. It helps to localize the key regions in the image that contribute to the classification decision and provides a visual understanding of the same through the overlaid heat maps. As referenced from the colormap, the regions in red are of higher importance compared to those in blue.

From a comparison of noisy and denoised samples, it is observed that the significant activations in the Grad-CAMs are recorded at the center and on the diffraction rings of the cell-lines. The noise in the images skews the activations by either diminishing them in regions of interest or by increasing activations outside those regions. This skew is however corrected by the denoising as shown in Fig. 2.

Again, Saliency Maps help to identify the differentiating regions of interest that have a significant role in determination of the class label of the images. From a comparison of saliency, it is observed that denoising suppresses the pseudo-important regions of interest that have been generated due to the noise in the image.
